# Second generation androgen receptor antagonist, TQB3720 abrogates prostate cancer growth *via* AR/GPX4 axis activated ferroptosis

**DOI:** 10.3389/fphar.2023.1110146

**Published:** 2023-01-20

**Authors:** Zhongqing Zhang, Tianlei Xie, Shun Zhang, Haoli Yin, Xuyu Zhang, Siyuan Zhang, Wei Chen, Ding Yu, Xuefeng Qiu, Wei Zhao, Hongqian Guo, Junlong Zhuang

**Affiliations:** ^1^ Department of Urology, Nanjing Drum Tower Hospital, The Affiliated Hospital of Nanjing University Medical School, Nanjing, China; ^2^ Nanjing Drum Tower Hospital Clinical College of Nanjing Medical University, Nanjing, China; ^3^ Institute of Urology Nanjing University, Nanjing, China; ^4^ School of Laboratory Medicine/Sichuan Provincial Engineering Laboratory for Prevention and Control Technology of Veterinary Drug Residue in Animal-Origin Food, Chengdu Medical College, Chengdu, China; ^5^ Chia Tai Tianqing Pharmaceutical Group Co., Ltd., Nanjing, China

**Keywords:** prostate cancer, androgen receptor, TQB3720, ferroptosis, GPx4

## Abstract

**Purpose:** Prostate cancer (PCa) poses a great threat to humans. The study aimed to evaluate the potential of TQB3720 in promoting ferroptosis to suppress prostate cancer, providing a theoretical basis for PCa therapy.

**Methods:** PCa cells and nude mice models were divided into TQB3720, enzalutamide (ENZ), and control groups. Sulforhodamine B assay, colony formation assessment, organoids culture system, and the CCK8 assay were used for detecting proliferation. Western blot assay was processed to detect the expression of androgen receptor (AR), ferroptosis, and apoptosis-related genes. Flow cytometry was applied to measure the intracellular ROS levels. ELISA was performed to determine the cellular oxidized glutathione (GSSG) and malondialdehyde (MDA) levels. RT-qPCR was conducted to detect the mRNA expression of genes in AR signaling. BODIPYTM™ 581/591 was processed for detection of intracellular lipid peroxidation levels. The interaction of AR with other translational factor complex proteins was explored using Co-immunoprecipitation (Co-IP), and the chromatin immunoprecipitation (ChIP) assay was performed to detect the binding of AR-involved translational complex to downstream genes promoter. Luciferase reporter assay was conducted to examine the translation activity of GPX4 promoter, and immunohistochemistry (IHC) was conducted to analyze the levels of c-MYC, Ki-67 and AR in TQB3720-treated cancer tissues.

**Results:** Here, we found TQB3720 inhibits the growth of prostate cancer *in vitro* and *in vivo*. TQB3720 treatment induced intracellular levels of GSSG and MDA significantly, by which hints AR antagonist caused ferroptosis-related cell death. Moreover, molecular evidence shown TQB3720 regulates downstream of AR signaling by binding AR resulting in inhibition of AR entry into the nucleus. Additional, we also proved that TQB3720 abrogates the interaction between AR and SP1 and leads to decrease GPX4 transcription.

**Conclusion:** TQB3720 promotes ferroptosis in prostate cancer cells by reducing the AR/SP1 transcriptional complex binding to GPX4 promoter. As a result, it is suggested to be a potential drug for clinic prostate cancer treatment.

## Introduction

Prostate cancer (PCa) is a malignant tumor of the genitourinary system, accounting for about 14.1% of malignant tumors in men (Sung et al., 2021). Early-stage patients mostly have no obvious symptoms, whereas late-stage patients may experience abnormal urination, abnormal urodynamics, blood in semen, erectile dysfunction, discomfort in the pelvic region, and bone pain (Litwin and Tan, 2017). Most patients are in the middle to the late stage at diagnosis. Therefore, it is important to develop novel drugs for countering against PCa (Sekhoacha et al., 2022).

In previous studies, AR was reported to transduce hormonal signals to regulates multiple prostate cancer events, proliferation, apoptosis, migration, invasion, and differentiation (Jacob et al., 2021; Westaby et al., 2022). As a translational factor, the transactivation function of AR can be regulated by Specificity Protein 1 (SP1) (Chen et al., 2008), the interaction between AR and SP1 stimulated the production of Vascular Endothelial Growth Factor (VEGF) (Eisermann et al., 2013) and Erythropoietin-Producing Hepatocellular carcinoma cell surface type-A receptor 3 (EPHA3) (Diao et al., 2018), which promotes PCa growth and progession. Thus, targeting the AR/SP1 translational complex showing therapeutic potential in prostate cancer (Wang and Ferrari, 2006; Yuan et al., 2010). In the past 30 years, huge number of anti-AR drugs have been developed and approved for different stages of prostate cancer, such as flutamide, bicalutamide, nilumet, and enzalutamide (Bassetto et al., 2016). First-generation AR antagonists have shortcomings, including shorter half-life and lower steady-state levels in the brain (Clement and Karen, 2022). Thus, the scientists develop the second-generation AR antagonists to address these shortcomings. Nowadays, TQB3720 has been considered as a second-generation AR antagonist (Chen et al., 2022). However, the molecular mechanisms of TQB3720 in PCa remains unexplored completely.

To uncover the potential mechanism of TQB3720 in PCa, we focused on ferroptosis. Emerging studies have shown that ferroptosis is involved in the development of prostate cancer. Ferroptosis is an iron-dependent and non-apoptotic form of cell death characterized by the accumulation of iron and ROS (Shen et al., 2021). However, iron homeostasis is essential in the development of prostate cancer, iron toxicity inhibits the growth of cancer cells (Raymonvil, 2017). Ferroptosis-mediating factors (SLC7A11, SLC3A2, GPX4) are aberrantly expressed in AR-resistant prostate cancer (Zaffaroni and Beretta, 2022). The induction of ferroptosis in tumor cells is expected to address drug resistance to conventional radiotherapy (Zhang et al., 2019; Zhou et al., 2019). TQB3720, a second-generation AR antagonist which is developed by Chia Tai Tianqing Pharmaceutical Group Co., Ltd., competes for binding to AR and causes its loss of function (Chen et al., 2022). Most importantly, this chemical is under the evaluation of the tolerance and pharmacokinetics in clinical trial phase 1 stage (ClinicalTrials.gov Identifier: NCT04853498). In this study, we investigated the potential molecular mechanisms of TQB3720 in ferroptosis of prostate cancer development using the *in vivo* and *in vitro* models.

## Materials and methods

### Cells and treatment

The prostate cancer cell lines (LNCap95 and 22RV1) were purchased from American Type Culture Collection (ATCC). Cells were cultured with RPMI-1640 medium (Gibco) containing 10% fetal bovine serum and incubated in a cell incubator (Thermo Fisher Scientific) with 37°C and 5% CO_2_. Moreover, inhibitors of multiple death pathways, including Ferrostatin-1 (Sigma-Aldrich), Liprostatin-1 (Sigma-Aldrich), Necrosulfonamide (Selleckchem), ZVAD-FMK (Selleckchem), and Chloroquine (Selleckchem), were used to detect the function of TQB3720 (TQB) and enzalutamide (ENZ). Synbio Technologies Co. Ltd. (Suzhou, China) synthesized mixture siRNAs (target AR and GPX4) and HA-AR while their transfection was conducted using the lipofectamine 2000 reagent (Invitrogen) as our previous reports (Zhao et al., 2014; Huang et al., 2019).

### Sulforhodamine B assay

Cells were treated with different concentrations of TQB3720 (0, 0.5, 1, 2, and 5 μM, Chia Tai Tianqing Pharmaceutical Group Co., Ltd.), while similar concentrations of Enzalutamide (ENZ, 0, 0.5, 1, 2, and 5 μM, Selleckchem) were used as a reference to clarify the dose-time-effect of TQB3720. After incubation for 24, 48, and 72 h, the plates were taken out, and 50 μL of 10% trichloroacetic acid (TCA) was added to each well before fixing for 1 h at 4°C. After fixation, the liquid in the wells was discarded, and the cells were washed 5 times with deionized water to remove the TCA. 100 μL of 0.4% SRB reagent (LMAI Bio Co. Ltd., China) was added to each well and kept at room temperature for 20 min. The liquid in the wells was discarded, and the unbound reagent was removed using 1% acetic acid and dried at room temperature. 150 μL of dimethyl sulfoxide (DMSO, Sigma-Aldrich) was then added and shaken for 10 min on a microplate shaker to lyse the cells (QB-9001, Qilinbeier Co. Ltd., China). The absorbance value (OD570) of each well was measured by a Microplate reader (Thermo Fisher Scientific) and used to calculate the effect of the drug on cell proliferation.

### Colony formation assay

LNCap95 and 22RV1 cells were inoculated in 6-well plates with a cell density of 500 cells/well. The experiments were divided into control, TQB3720 (5 µM), and GA+ENZ (5 µM) groups. After 48 h of treatment, the cells were washed 3 times with PBS buffer and stained with crystal violet (Sangon Biotech, China). Colonies formed in each group were observed, and the number of colonies with more than 10 cells was counted by photographing them under a microscope (Olympus). Colony formation rate was calculated as previous reports (Liu et al., 2021; Sun et al., 2022).

### Organoids culture system

The prostate cancer tissue was collected from Department of Urology, Nanjing Drum Tower Hospital, The Affiliated Hospital of Nanjing University Medical School with patients written informed consents. This study has been approved by Ethics Committee of Nanjing University Medical School. The collected prostate cancer tissues were clipped on ice and resuspended by adding collagenase (Invitrogen). After digestion, the cells were filtered and the filtrate was added to DMEM/F12 (Gibco) to terminate the digestion. Centrifugation was then used to separate the supernatant. Erythrocyte lysate was used to resuspend the cells. After centrifugation, the supernatant was removed and DMEM/F12 was added to resuspend cells. After centrifugation, the supernatant was removed, cells were counted, and matrigel (Gibco) was mixed. Samples were dropped in each well of the plate, culture dishes were placed, and martrigel was coagulated. 500 nM of A83-01 medium (STEMCELL technologies) was added to each well, and the cells were incubated. The sample plates were then digested for 15–25 min and uniformly distributed in microtiter plates. The sample plates were incubated for 22–26 h, then replaced with the drug-containing medium. To maintain the growth conditions after spreading, the plates were incubated for 24 h. This was done to mimic the conditions during *in vivo* drug delivery.

### Western blot assay

The cells were inoculated in 100 mm culture dishes and incubated for 48 h after drug treatment. After sufficient lysis of the cells, the supernatant was collected by centrifugation. The protein content was determined using the BCA kit (Sigma-Aldrich). SDS-PAGE electrophoresis was performed to separate proteins with different relative molecular masses, which were then transferred onto PVDF membranes (Thermo Fisher Scientific). The proteins were blocked with 5% skim milk for 2 h, washed 3 times with TBST, and incubated with primary antibodies (GPX4, AR, FKBP51, PSA, LK2, S100P, TMPRSS2, Bax, caspase-3, Bcl-2, GPX1, AR, PSA, 1:1000, all purchased from Abcam) overnight at 4°C. PVDF membranes were washed with TBST, and peroxidase-labeled secondary antibody (1:10000, Abcam) was added, incubated for 2 h at room temperature, and washed 4 times with PBST. The proteins were visualized by a DAB immunohistochemistry color development kit (Sangon Biotech, China) and photographed using a gel imaging analysis system (Thermo Fisher Scientifi). Relative protein levels were calculated by ImageJ as previous report (Sun et al., 2022).

### Flow cytometry assay

The cells were cultured for 6 h after TQB3720 treatment. DCFH-DA staining solution and serum-free culture medium were diluted at 1:1000, and 2 mL of the working solution was added to cell-containing wells. The cells were then incubated for 20 min at 37°C. The cells were digested using trypsin, collected, and resuspended in PBS buffer after 3 washes with a serum-free culture medium. Intracellular ROS levels were measured with an Accuri C6 flow cytometer (BD Accuri C6) with an excitation wavelength of 488 nm and an emission wavelength of 525 nm.

### ELISA assay

Cellular GSSG and MDA levels were measured by ELISA assay. The standard solutions were diluted, and the standard curve was plotted following the ELISA kit (Sigma, Shanghai) manufacturer’s instructions. 40 μL of sample and 10 μL of biotin-labeled antibody were added to each ELISA plate well. 50 μL of standard enzyme reagent was added to each well except the blanks. The plate was sealed and incubated at 37°C for 30 min. The sealing membrane was removed, and the liquid was discarded and dried. Each well was filled with the washing solution and allowed to stand for 30 s. 50 μL of reagent A and 50 μL of reagent B were added to each well, gently shaken, and mixed. The cell mixture was then incubated, and the samples were developed at 37°C for 10 min in darkness. 50 μL of termination solution was used to terminate the reaction and the results were detected using a microplate reader (Bio-Rad).

### RT-qPCR assay

RT-qPCR assay was conducted to detect the expression of AR signaling-related genes (AR, FKBP51, PSA, LK2, S100P, TMPRSS2), ferroptosis-related gene (GPX4), and apoptosis-related genes (Bax, caspase-3, Bcl-2). The cells were inoculated in cell culture flasks with 1 × 10^6^ cells. After 24 h, TQB3720 was introduced in doses of 0, 2, and 5 μM, and the treatment was terminated after 48 h. The cells were collected, and total RNA was extracted by TRIzol reagent (Invitrogen) and reverse transcribed into cDNA using a reverse transcription kit (QIAGEN). The amplification conditions included 95°C for 3 min, 95°C for 15 s, 58°C for 30 s, 72°C for 30 s, and 35 cycles. A fluorescence real-time quantitative PCR kit (Thermo Fisher Scientific) was used for RT-qPCR detection with primers synthesized by Invitrogen (Table 1), and GAPDH was used as an internal reference. All the used primers were purchased from Sangon (Shanghai, China). Ct values were analyzed and processed as 2^−ΔΔCT^ (Ren et al., 2021).

**TABLE 1:** Primers sequence for qRTPCR.

Gene symbol	Forward	Reverse
AR	CAG​TGC​TGT​ACA​GGA​GCC​GAA	CTT​CAC​CGA​AGA​GGA​AAG​GGC​A
FKBP5	GGC​TGA​AGG​GTT​AGC​GGA​G	GCT​GTG​GGG​CTT​TCT​TCA​TTG
PSA	CTC​AGG​CCA​GGT​GAT​GAC​TC	GTC​CAG​CAC​ACA​GCA​TGA​AC
KLK2	AGT​CAT​GGA​TGG​GCA​CAC​TG	CTC​TGG​CCT​GTG​TCT​TCA​GG
S100P	ATC​ACG​TCT​GCC​TGT​CAC​AA	CAC​TTT​TGG​GAA​GCC​TGG​GA
TMPRSS2	ACA​CAC​CGA​TTC​TCG​TCC​T	TGG​CCT​ACT​CTG​GAA​GTT​CA
GPX4	AGC​AAG​ATC​TGC​GTG​AAC​GG	TGG​AGA​GAC​GGT​GTC​CAA​AC
Bax	GAGCAGCCCAGAGGCG	GGACGCATCCTGAGGCAC
Bcl2	AAA​AAT​ACA​ACA​TCA​CAG​AGG​AAG​T	CCT​TGG​CAT​GAG​ATG​CAG​GA
Caspase-3	TGC​TAT​TGT​GAG​GCG​GTT​GT	TCA​CGG​CCT​GGG​ATT​TCA​AG
Beta-actin	GAG​CTA​CGA​GCT​GCC​TGA​CG	CCT​AGA​AGC​ATT​TGC​GGT​GG

### Detection of intracellular lipid peroxidation levels

The cells were grown in 24-well plates, and when the cell density grew to about 60%–70%, the drug was added for 7 h. 1 μmol/L BODIPYTM 581/591 solution was added into the wells with the cells and incubated for 1 h at 37°C. Random photographic recordings were processed under fluorescence microscopy. The blue color represented the hochest-stained nuclei, while the red indicated the lipid peroxidation level.

### CCK8 assay

The effect of TQB on cell proliferation was detected using the CCK8 kit (DOJINDO, Japan). The cells were inoculated in 96-well plates at a density of 8 × 10^3^ cells per well and incubated at 37°C in a 5% CO_2_ incubator. After culture apposition, the culture medium was discarded, and the cells were treated with TBQ, HA-AR, and siGPX4 for 48 h. The original culture medium was discarded and 100 μL of culture medium containing 10% CCK8 reagent was added to each well and incubated for 2 h at 37°C. The absorbance value of each well was measured at 450 nm using a microplate reader (Bio-Rad, USA).

### Co-IP assay

After 24 h of treatment, the cells were collected, lysed with 0.5 mL of lysis solution, and then centrifuged for 30 min in an ice bath to obtain the supernatant for immunoprecipitation assay. Briefly, the assay followed our previous report (Zhao et al., 2014). The antibodies (anti-AR, cat.no. 06-680, Sigma, Shanghai; anti-SP1, cat.no. ab231778, Abcam, Shanghai; anti-Tubulin-α, cat.no. ab52866, Abcam) for immunoprecipitation were added to the supernatant and incubated overnight at 4°C. Agarose beads protein A-binding antibodies were added and incubated at 4°C for 4 h. The precipitates were washed 4 times with lysate, each time for 10 min. The precipitate was added to 20 μL of 2×SDS protein buffer, denatured by boiling for 10 min, and subjected to SDS-PAGE electrophoresis.

### ChIP assay

After 48 h of treatment, cells were collected and lysed. ChIP experiments were then performed to show DNA sequences (including GPX4 promoter) bound to AR or SP1 proteins. The experiments were performed using the ChIP kit (cat.no. ab185913, Abcam) following the manufacturer’s instructions. DNA-binding proteins were cross-linked to DNA *in vivo* using formaldehyde. After chromosomal separation, the sheared DNA fragments were associated with the binding proteins. The Specific antibodies (anti-AR, cat.no. 06-680, Sigma, Shanghai; anti-SP1, cat.no. ab231778, Abcam, Shanghai; anti-Tubulin-α, cat.no. ab52866, Abcam) were used to precipitate and to isolate the complex containing targeted proteins and its binding DNAs. The DNA was released by reverse cross-linking, and the protein was digested. RT-qPCR was performed to detect GPX4 promoter, and western blot was conducted to detect targeted proteins.

### Dual-luciferase reporter assay

The promoter (−500 bp, 50 bp) of GPX4, recorded as RefSeq NM_00208 in NCBI database, was constructed into pGLO vector. After 12 h transfection with GPX4 promoter (pGLO system, cat.no.2920, Promega, Madison, USA), the cells were incubated with 5 μM TQB7320 or 5 μM ENZ for 48 h. Afterwards, the cells were lysed by using passive lysis buffer according to the protocols of Promega kit (cat.no. J3081, Promega). The luciferase activity was determined by using Promega GloMaxTM 20/20 instrument. Luciferase assay substrate was added into the supernatant of cell samples. After mixing completely and measuring immediately as fluorescence intensity I, they were added with the “stop & glo” solution to stop the reaction and the corresponding luciferase activity was recorded as fluorescence intensity II. The relative luciferase activity was calculated as the ratio of fluorescence value II to I.

### Preparation and grouping of mouse models of prostate cancer

LNCap95 and 22RV1 cells were cultured in 10% fetal bovine serum, RPMI 1640 medium at 37°C, and 5% CO_2_. The cells were then inoculated subcutaneously on the back shoulder of mice at the logarithmic growth stage, and then randomly divided into several groups: TQB3720 (0 mg/kg body weight), TQB3720 (20 mg/kg body weight), and TQB3720 (40 mg/kg body weight). The animal experiments were approved by the biomedical ethics committee of Chengdu Medical College. The initiating day was recorded as the 0 days. The tumor size of mice was measured every 3 days. Finally, the cancerous tissues were collected for further analysis.

### IHC assay

The assay was conducted using the ready-to-use immunohistochemistry kit (Biolab, cat.no. GS4974, Bejing, China). Following the manufacturer’s instructions, the negative control group of the experiment was substituted with phosphate buffer solution (PBS) for the primary antibody. Tissue sections were incubated in an oven at 70°C for 2 h, dewaxed in xylene 3 times, and hydrated in alcohol. The sections were then soaked in a 3% H_2_O_2_ mixture, rinsed with PBS buffer and distilled water in turns, placed in 0.01 mol/L raffinate buffer, transferred to an autoclave (120°C∼140°C) and heat-repaired antigen for 10 min. The sections were incubated with primary antibody in a wet box for 20 min overnight at 4°C. The samples were kept at room temperature, rinsed, and a secondary antibody was added, and incubation was continued for 30 min in a wet box at 37°C. The sections were washed 3 times with PBS and developed with DAB. Mayer’s hematoxylin was re-stained for 5 s, and the films were sealed with neutral gum. Images were taken immediately using a LSM 780 microscope (Zeiss, Germany), LSM 880 Fast Ariyscan, or SP8 microscope (Leica, Germany).

### Statistical analysis

All the data were analyzed using SPSS 20.0 statistical software (IBM, *Armonk, New York, USA*). Measurement data were expressed as mean ± standard deviation ( $\bar{\;x}$ ± s). A *t*-test was used to compare the means of multiple groups. *p* < 0.05 was considered statistically significant.

## Results

### TQB3720 inhibited the growth of prostate cells

**FIGURE 1:**
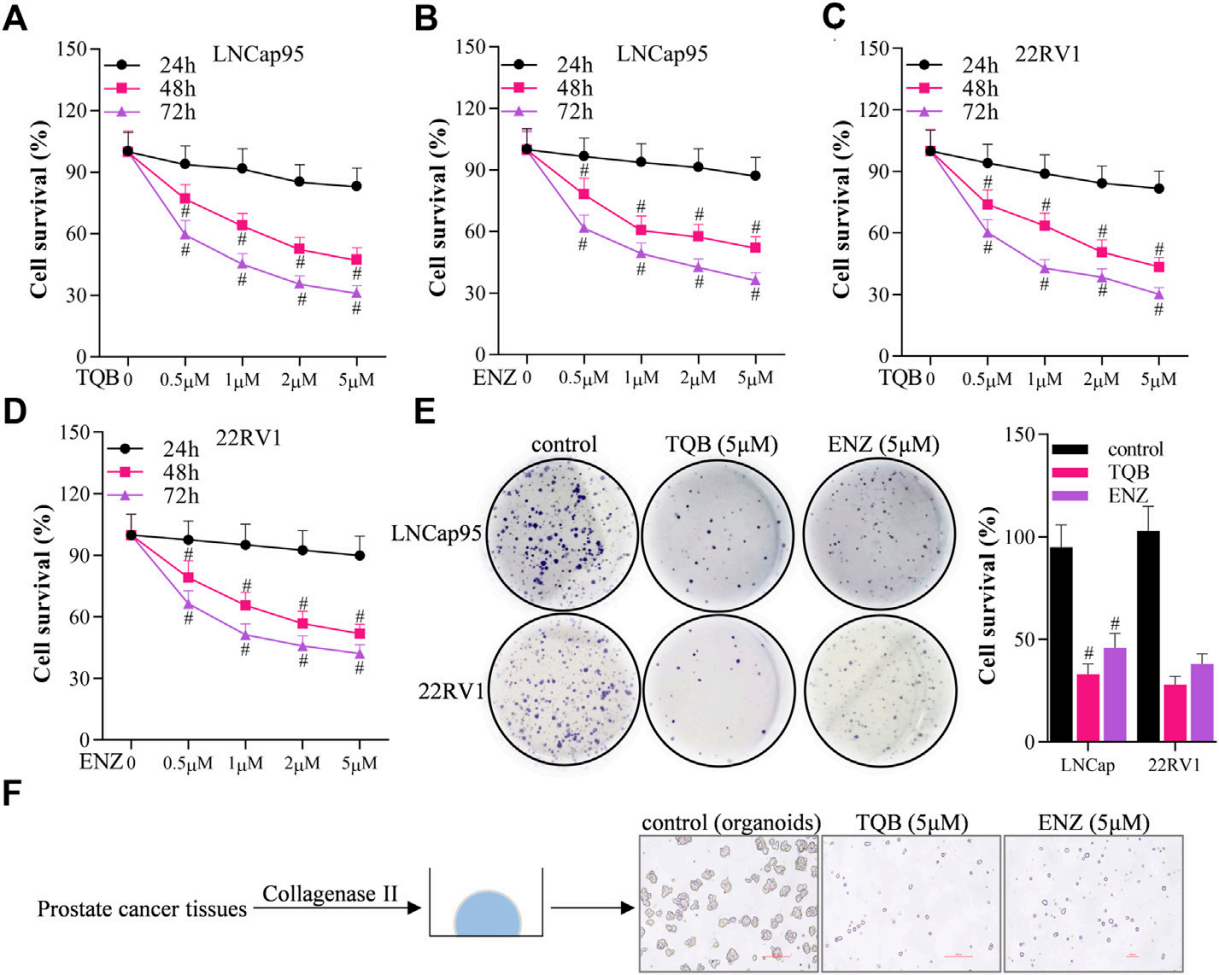
TQB3720 inhibited the growth of prostate cells **(A–D)** Sulforhodamine B assays were conducted to detect the effect of TQB3720 on the proliferation of LNCap95 and 22RV1 cells, ^#^ *p* < 0.01 vs. 24 h. **(E)** Effect of TQB3720 on colony formation of LNCap95, 22RV1 cells, ^#^ *p* < 0.01 vs. 24 h. **(F)** Organoid assay showing the effect of TQB3720 on organoid formation of clinic human prostate cancer primary cells.

Sulforhodamine B assays were used to determine the effects of TQB3720 and ENZ on the proliferation of LNCap95 and 22RV1 cells to determine the dose-time effect of TQB3720. Here, both TQB3720 and positive control, ENZ inhibited the survival rate of LNCap and 22RV1 cells. Importantly, the effects of both drugs were dose-time dependent (Figures 1A–D). 5 µM TQB3720 and ENZ had the most significant effects and were used for the following experiments. Effects of TQB3720 on the proliferation of LNCap95, 22RV1 cells were determined by assessing colony formation. TQB3720 and ENZ significantly decreased the survival rate of both cell lines. Compared with the ENZ group, the inhibitory effect of TQB3720 was more evident, although there were no statistically significant differences (Figure 1E). Moreover, an organoids culture system was used to detect the effect of TQB3720 on organoid formation from prostate cancer tumors. It was observed that TQB3720 inhibited the growth of prostate tissue (Figure 1F), suggesting the inhibitory effects of TQB3720 on the growth of prostate cells.

### TQB3720 induces ferroptosis in prostate cancer cells

**FIGURE 2:**
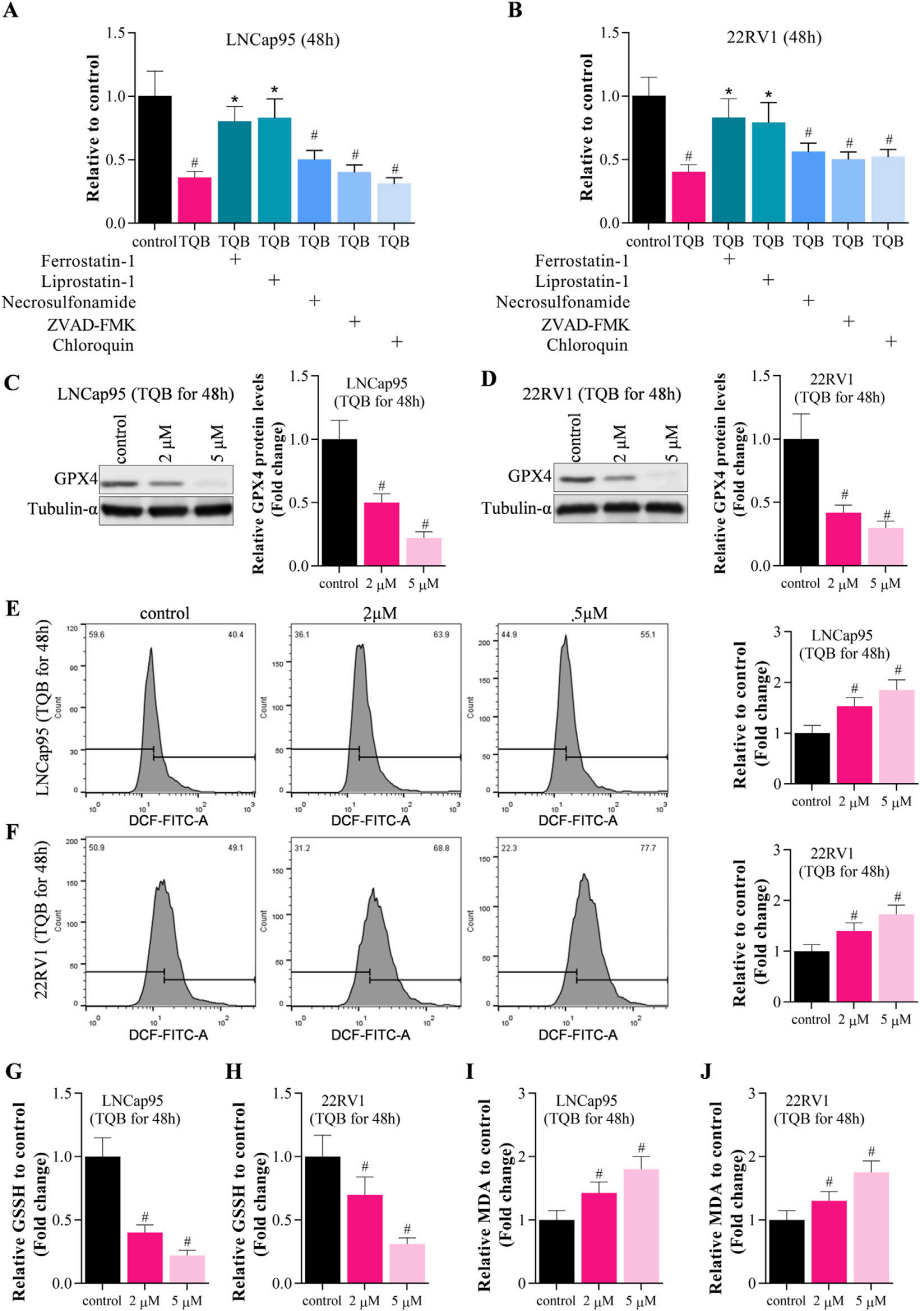
TQB3720 induced ferroptosis of prostate cancer cells. **(A,B)** The proliferation of LNCap95 and 22RV1 cells significantly decreased following TQB3720 incubation for 48 h, whereas ferrostatin-1 and liprostatin-1 abolished this effect, ^#^ *p* < 0.01 vs. control, *p < 0.01 vs. TQB3720 (5 μM) **(C,D)** Western blot showed effects of TQB3720 on expression of ferroptosis marker GPX4, ^#^ *p* < 0.01 vs. control. **(E,F)** Flow cytometry assay showed the effects of TQB3720 on ROS levels using DCFH-DA probes, ^#^ *p* < 0.01 vs. control. **(G,H)** ELISA results showed the effects of TQB3720 on GSSG levels in cancer cells, ^#^ *p* < 0.01 vs. control. **(I–J)** ELISA assay proved that the effects of TQB7320 on MDA levels in cancer cells, ^#^ *p* < 0.01 vs. control.

Above data demonstrated that TQB3720 inhibits the growth of prostate cells. To uncover the detailed effect of 2nd generation of AR antagonist on PCa cells, Sulforhodamine B assays were conducted to determine the effect of TQB3720 and other inhibitors such as ferrostatub-1, liprostatin-1, necrosulfonamide, ZVAD-FAK, and chloroquine on the proliferation of LNCap95 and 22RV1 cells. After 48 h of TQB3720 treatment, the proliferation of LNCap95 and 22RV1 cells significantly decreased, while the inhibition was rescued by ferrostatin-1 and liprostatin-1, necrosulfonamide, ZVAD-FMK, and chloroquine failed to rescue PCa cells (Figures 2A, B). The results suggest that ferroptosis is a major cell death when TQB3720 treated PCa cells. Western blot assay indicated that 2 and 5 µM of TQB3720 inhibited GPX4 expression in LNCap95 and 22RV1 cells (Figures 2C, D). The results hint that TQB3720 induces iron-dependent death in PCa cells.

GPX4 impairs lipid peroxide toxicity through its catalytic activity and maintains membrane lipid bilayer homeostasis, and inactivation of GPX4 induces Reactive Oxygen Species (ROS) accumulation and triggers iron-dependent death (Kang et al., 2022; Yang W et al., 2022). In Figures 2E, F, data shown that 2 and 5 µM TQB3720 treatment increased the intracellular level of ROS significantly. Compared with the control, ELISA assay also indicated that cellular oxidized glutathione (GSSG) and malondialdehyde (MDA) levels were significantly upregulated by TQB3720 treatment in PCa cells (Figures 2G–J). Taken together, data in Figure 2 demonstrated that TQB3720 induces ferroptosis in prostate cancer cells.

### TQB3720 activates ferroptosis *via* the AR-GPX4 axis

**FIGURE 3:**
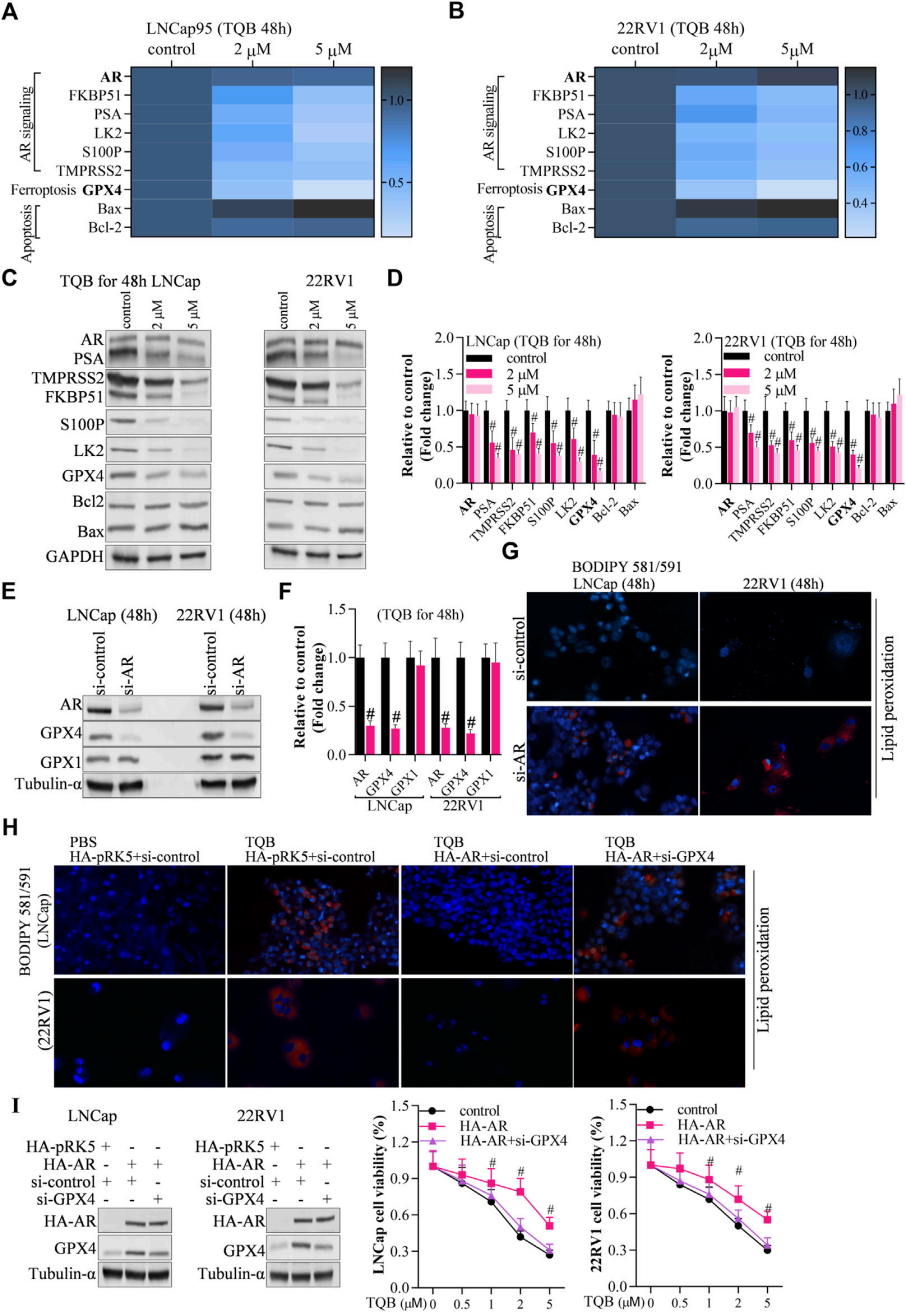
TQB3720 induced ferroptosis *via* AR-GPX4 axis **(A,B)** RT-qPCR detected the effects of on expression of genes in AR signaling pathway, ferroptosis pathway, and apoptotic pathway, ^#^ *p* < 0.01 vs. control. **(C,D)** Western blot examined the effects of on expression of genes in AR signaling pathway, ferroptosis pathway, and apoptotic pathway, ^#^ *p* < 0.01 vs. control. **(E,F)** Western blot determined the expression of AR and GPX4 after knockdown of AR, ^#^ *p* < 0.01 *vs.* si-control. **(G)** The BODIPY 581/591 assay revealed the effects of AR knockdown on lipid peroxidation. Blue indicates the hochest stained nuclei, and red indicates lipid peroxidation levels. **(H)** The cells were transfected with HA-AR for 24 h after knocking down GPX4 for 48 h, and then they were incubated with 5 μM TQB3720 for another 48 h. The BODIPY 581/591 assay was used to determine the lipid peroxidation levels indicated by hochest-stained nuclei in blue and lipid peroxidation in red. **(I)** The cells were transfected with HA-AR for 24 h after knocking GPX4 down for 48 h and then incubated with 5 μM TQB3720 for another 48 h, and then, MTT assay was used to detect the cell proliferation, ^#^ *p* < 0.01 vs. control.

To identify the underlying molecular mechanism of TQB3720 induced-ferroptosis, the expression of AR signaling-related genes (AR, FKBP51, PSA, LK2, S100P, TMPRSS2), ferroptosis-related genes (GPX4), and apoptosis-related genes (Bax, caspase-3, Bcl-2). The expression levels of FKBP51, PSA, LK2, S100P, TMPRSS2, and Bcl-2 GPX4 were downregulated by 2 and 5 µM of TQB3720. The apoptosis-related genes, caspase-3 was also downregulated, and Bax was up-related in TQB3720 groups compared with the controls (Figures 3A–D). Moreover, the knockdown of AR abrogates GPX4 expression, but no effect on GPX1 (Figures 3E, F). The BODIPY 581/591 assay revealed the knockdown of AR-induced lipid peroxidation (Figure 3G). In Figure 3H, TQB3720 induced PCa cells lipid peroxidation. Exogenous AR inhibited intracellular lipid peroxidation, while knockdown of GPX4 induced PCa cells lipid peroxidation (Figure 3H). When Furthermore, HA-AR increased cell viability, while si-GPX4 had inhibitory effects. Cell viability gradually decreased with increasing TQB3720 concentration (Figure 3I). Our results indicate that TQB3720 induces ferroptosis *via* the AR-GPX4 axis.

### TQB3720 inhibits downstream of AR signaling by binding AR and inhibiting AR entry into the nucleus

**FIGURE 4:**
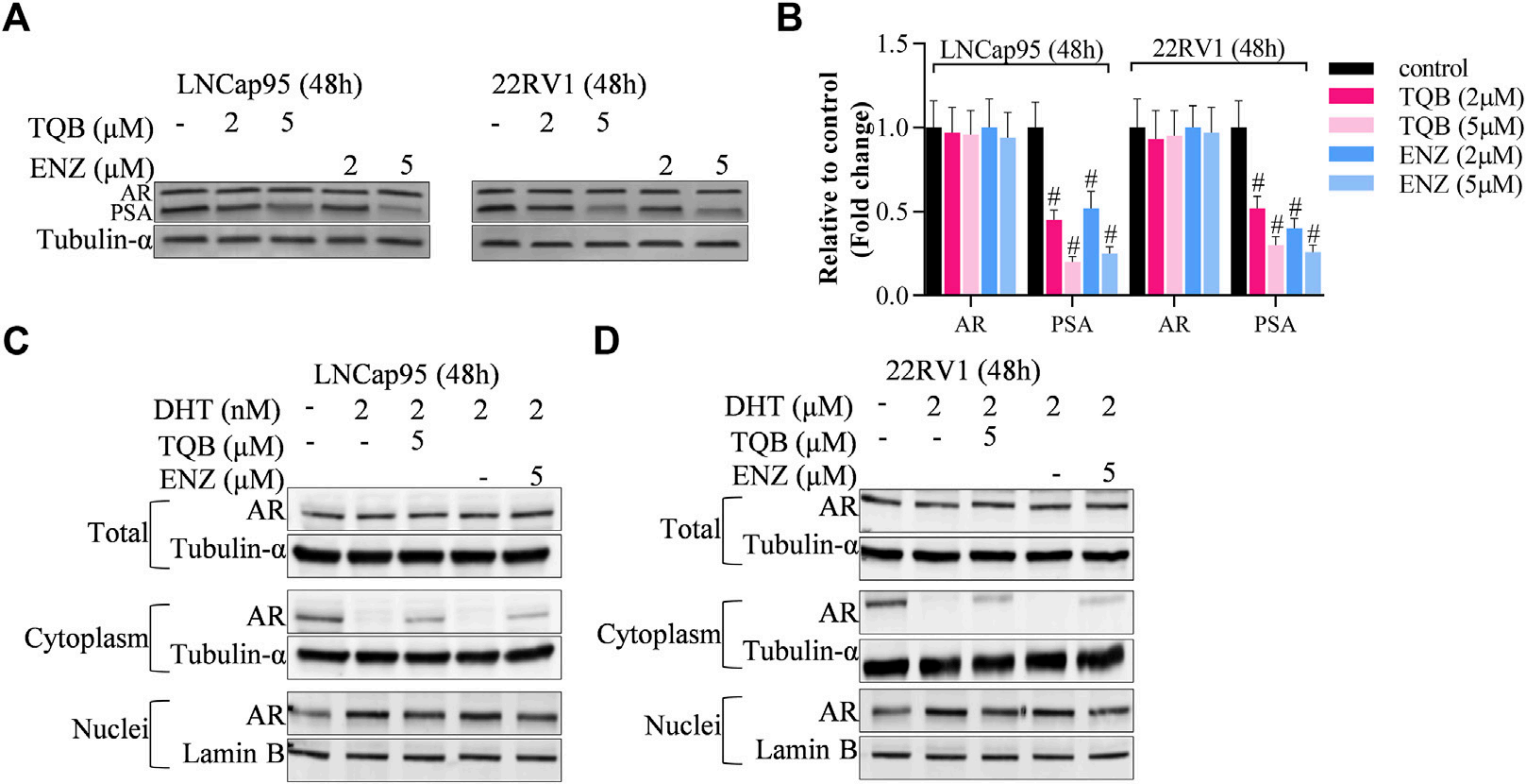
TQB3720 inhibited downstream of AR signaling by inhibiting AR entry into the nucleus **(A, B)** Western blot examined the effects of TQB3720 and ENZ on PSA expressions, and the protein band density was determined using ImageJ software, ^#^ *p* < 0.01 vs. control (absence of any indicated chemicals). **(C,D)** Western blot examined the effects of TQB3720 and ENZ on AR entry into nuclei from cytoplasm, and the protein band density was determined using ImageJ software, ^#^ *p* < 0.01 vs. control (absence of any indicated chemicals).

As a second-generation AR antagonist, above data proved that TQB3720 alleviates AR downstream genes expression. Here, we found PSA was significantly downregulated by TQB3720 and ENZ treatment, while AR expression without change (Figure 4A). Then, the cells were incubated with DHT for 2h, followed by TQB3720 and ENZ stimulation for the next 48 h. They were collected for nuclei-cytoplasm separation, and the contents of AR in the nucleus and plasma of the cells were detected using western blot. TQB3720 and ENZ inhibited DHT-mediated promotion of AR entry into the nucleus (Figures 4B–D). Therefore, TQB3720 did not affect AR protein levels but it suppressed downstream of AR signaling by inhibiting AR entry into the nucleus (Figures 4C, D). This mechanism indicates that administration of TQB7320 does not develop drug resistance.

### TQB3720 impairs GPX4 expression by inhibiting AR binding to SP1

**FIGURE 5:**
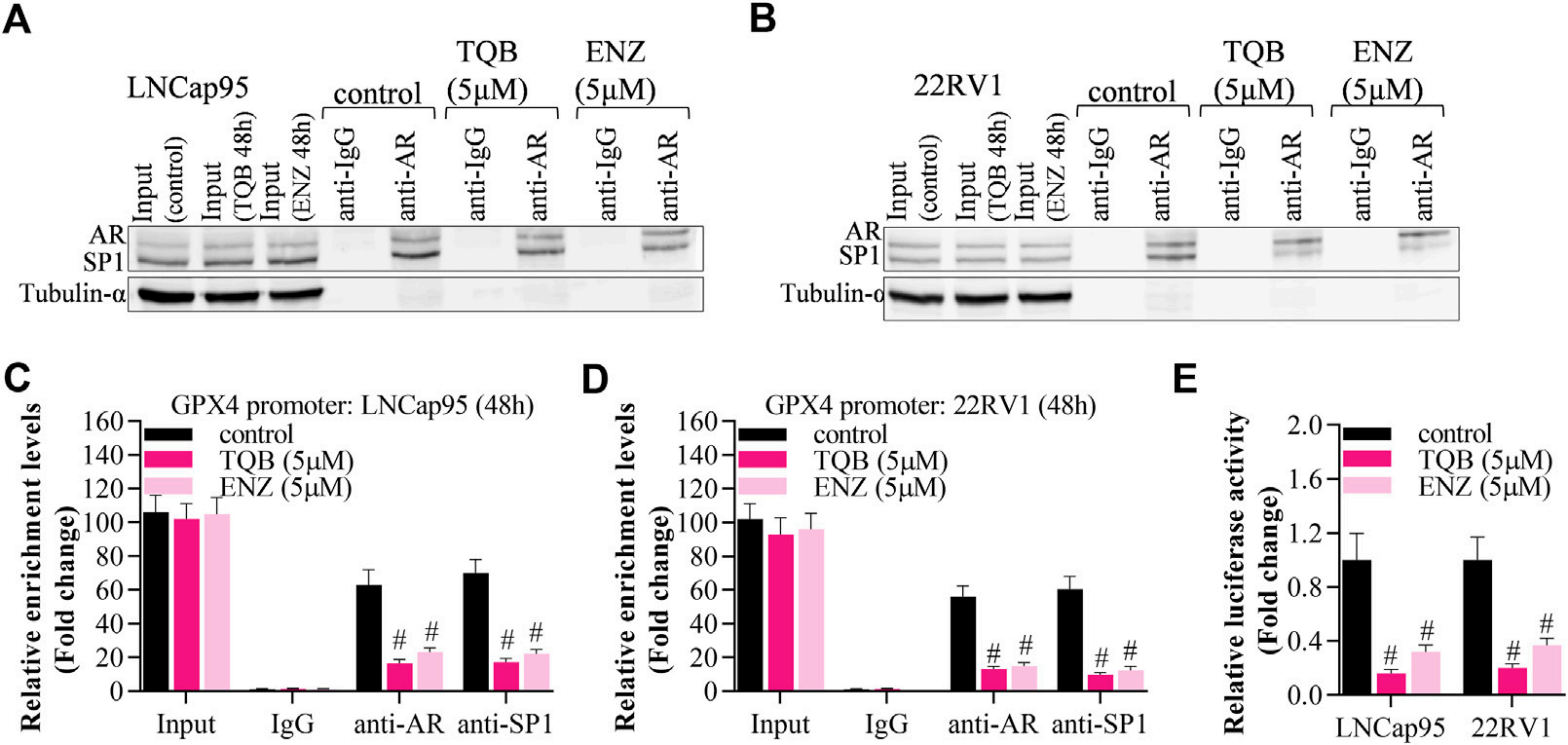
TQB3720 impaired GPX4 expression by inhibiting AR binding to SP1 **(A, B)** Co-IP assay showed the effect of TQB3720 on the interaction between AR and SP1 in LNcap95 and 22RV1. **(C, D)** RT-qPCR detected content of AR- or SP1-binding GPX4 promoter after performing ChIP assay, ^#^ *p* < 0.01 vs. control. **(E)** Luciferase reporter assay analyzed the impacts of TQB7320 on luciferase activity of GPX4 promoter, ^#^ *p* < 0.01 vs. control.

To identify whether TQB3720 disturb the AR/SP1 transcriptional factor complex in PCa cells, co-IP, ChIP and Luciferase reporter assays were performed. In both LNCap95 and 22RV1, an interaction between AR and SP1 was observed. However, this interaction was diminished after incubation of TQB3720 or ENZ (Figures 5A, B). By using anti-AR or anti-SP1, the ChIP data also confirmed that TQB3720 and ENZ groups showed less content of immunoprecipitated GPX4 promoter than in the controls (Figures 5C, D). Additionally, luciferase reporter assay showed that TQB3720 impaired GPX4 expression. The ChIP as well as RT-qPCR showed that TQB3720 inhibited the binding of AR/SP1 to the PSA promoter (Supplementary Figure S1). The highlighted results confirmed our speculation that TQB3720 decreases GPX4 expression by inhibiting the interaction between AR and SP1.

### TQB3720 suppresses the growth of prostate cancer cells *in vivo*

**FIGURE 6:**
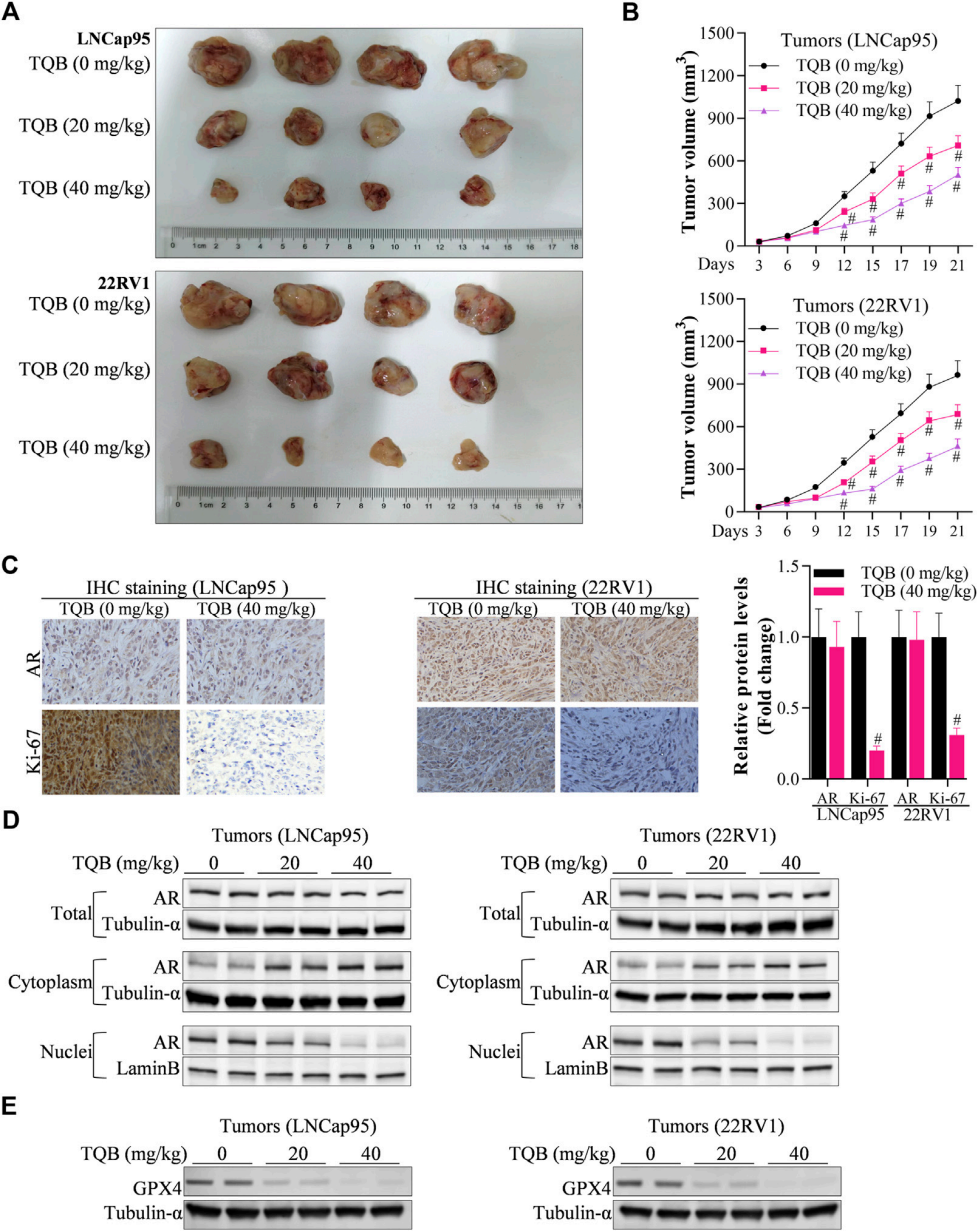
TQB3720 inhibited PCa carcinogenesis. **(A, B)** The tumor volume in each group of nude mice, ^#^ *p* < 0.01 vs. TQB3720 (0 mg/kg). **(C)** IHC examined the influences of TQB7320 on the expression levels of AR, Ki-67, ^#^ *p* < 0.01 vs. TQB3720 (0 mg/kg). **(D)** Western blot showed the impacts of TQB7320 on content of AR proteins in the nucleus or cytoplasm tumors from nude mice. **(E)** Western blot examined the effects of TQB7320 on expression of GPX4 protein in tumors.

The effect of TQB3720 on prostate cell tumor growth *in vivo* was verified through xenograft experiments. Compared with control group (TQB3720, 0 mg/kg), 20 and 40 mg/kg of TQB3720 significantly inhibited tumor growth (Figures 6A, B). Additionally, 40 mg/kg TQB3720 downregulated Ki-67 and c-MYC expression (Figure 6C). Heterogeneous tumor tissues were collected for nucleoplasmic separation, and the levels of AR proteins in the nucleus and plasma were detected by western blot. The results showed that AR entry into the nucleus was inhibited by 40 mg/kg TQB3720 (Figure 6D). Moreover, GPX4 expression was downregulated by 40 mg/kg TQB3720 *in vivo* (Figure 6E). The *in vivo* results were similar with those obtained *in vitro,* showing that TQB3720 impairs GPX4 expression and inhibits AR entry into the nucleus.

## Discussion

Prostate cancer, especially metastatic PCa, poses a great threat to humans worldwide (Raju et al., 2022). Therefore, developing effective and safe anti-cancer agents is critical to prolong the life of patients, reduce death rates, and improve survival. Traditional androgen deprivation therapy (ADT), which includes surgical and pharmacologic deprivation, improves the prognosis of PCa patients by lowering serum testosterone levels and inhibiting the androgen pathway (Cho et al., 2003). However, after 18–24 months of ADT, almost all PCa patients evolve into destructive-resistant prostate cancer (Oudard et al., 2014). Therefore, AR antagonists that act directly on the androgen receptor (AR) are critical in PCa treatment. The first-generation AR antagonists, such as bicalutamide and flutamide, have shown a low affinity for the receptor, poor antimutagenic ability, and hepatotoxic adverse effects in clinical applications (Soloway et al., 2000). Although second-generation AR antagonists have partially solved these problems, they are associated with drug resistance (Semenas et al., 2012). In this study, TQB3720 promoted ferroptosis in prostate cancer cells by alleviating the AR/SP1 transcriptional complex. It is suggested to be a potential drug for prostate cancer treatment, with lower drug resistance than ENZ.

During androgen deficiency, the cells develop an adaptive response by either allowing AR mutation or proliferation (Wang, 2009). AR regulates other signaling pathways to achieve the self-activating process. The transcriptional activity of AR is usually activated by androgen receptor signaling (Cronauer et al., 2003). Thus in depot-resistance diseases, there is a strong selective pressure to maintain the AR control signaling pathway (Clement and Karen, 2022). In this study, the expressions of FKBP51, PSA, LK2, S100P, TMPRSS2, GPX4, and Bcl-2 were downregulated by TQB3720 as well as caspase-3. As a result, TQB3720 improved apoptosis and inhibited AR signaling. Moreover, TQB3720 regulates AR downstream signaling by inhibiting AR entry into the nucleus, which is unique compared with first and second-generation AR antagonists. Moreover, the results from this study verified that AR binds to SP1. As reported in a previous study, aberrant expression and activation of SP1 protein in tumor tissues regulate tumor proliferation, angiogenesis, and metastatic ability (Yuan et al., 2005). Our results suggest that by inhibiting AR binding to SP1, TQB3720 is effective in prostate cancer. Based on the data from the clinical trial database (https://www.clinicaltrials.gov), TQB3720 has been investigated in numerous advanced cancers. Moreover, data from the global data database (https://pharmacn.globaldata.com) indicate that TQB3720 has been used in cancers, such as non-small cell lung cancer, gastric cancer, and cancer of the gallbladder. TQB3720, therefore, exhibits potential as a clinical prostate cancer treatment.

To clarify the molecular mechanisms of TQB3720 in the suppression of prostate cancer progression, we examined the effects of TQB3720 on ferroptosis. Ferroptosis refers to iron-dependent cell death caused by the excessive accumulation of ROS and lipid peroxides (Latunde-Dada, 2017). Highly malignant cancer cells have an innate susceptibility to ferroptosis, and thus induction of ferroptosis may be a new approach to cancer therapy (Mga et al., 2022). In this study, to evaluate the potential of TQB3720 to promote ferroptosis in prostate cancer, molecular mechanisms of TQB3720 were studied using *in vivo* and *in vitro* models. TQB3720 inhibits the growth of prostate cells and induces ferroptosis in prostate cancer cells. The level of ROS was higher in TQB3720 groups. TQB3720 treatment also significantly increased the levels of GSSG and MDA.

Moreover, ENZ was used as a reference for TQB3720, and *in vitro* experiments confirmed the non-inferiority of TQB3720 compared with ENZ, suggesting that TQB3720 maybe be a potential drug for prostate cancer treatment. GPX4 is the most potent defense against ferroptosis (Chang et al., 2022). In this study, TQB3720 induced ferroptosis by impairing GPX4 expression. Several therapeutic oncology regimens are highly susceptible to GPX4 inhibition, highlighting the importance of GPX4 inhibitors in targeting ferroptosis in cancer (Zhan et al., 2022). However, most inhibitors are associated with poor pharmacological properties in animal models, limiting their potential for clinical translation. The development and optimization of GPX4-targeted drugs with improved pharmacokinetics and selectivity remain a major challenge to the use of GPX4 inhibitors for cancer treatment (Mtca et al.). Thus, available and *in vivo* stable anticancer drugs with GPX4 inhibitory activities may provide an alternative approach. Previously, it was found that inhibition of GPX4 activity and expression activated ferroptosis and effectively inhibited tumor progression (Zhan et al., 2022). Ferroptosis is regulated by GPX4 and iron transport regulatory proteins (Song et al., 2020). Consistent with these reports, our study showed that TQB3720 suppressed GPX4 expression and GSSG levels while increasing the MDA levels. The cellular redox homeostasis factor GSSG and the lipid peroxidation product MDA are essential in maintaining cellular redox homeostasis (Wong and Bruice, 2012). The main manifestations of ferroptosis in biochemical metabolism are ionized iron deposition leading to membrane lipid peroxidation and excessive oxidative stress that impairs intracellular redox homeostasis, reducing antioxidant capacity and increasing intracellular lipid reactive oxygen species, ultimately leading to oxidative cell death (Yang Z et al., 2022).

In conclusion, TQB3720 promotes ferroptosis in prostate cancer cells by alleviating the AR/SP1 transcriptional complex. TQB3720-based cancer therapy is expected to bypass the drawbacks of conventional therapies mediated by apoptosis due to its ability to induce natural non-apoptotic forms. It is, therefore, a potential drug for prostate cancer treatment, with equivalent efficacy as ENZ. In future, we shall explore the clinical application of TQB3720 and identify strategies for improving the effectiveness and safety of the drug.

## Data Availability

The original contributions presented in the study are included in the article/Supplementary Material, further inquiries can be directed to the corresponding authors.

## Supplementary material

SUPPLEMENTARY FIGURE S1 RT-qPCR determined the effects of TQB3720 on the binding of AR/SP1 to PSA promoter after conducting ChIP assay. **(A,B)** The cell lines were incubated with TQB7320 and ENZ for 48 h, we used RT-qPCR to detect content of AR- or SP1-binding PSA promoter after performing ChIP assay, ^#^ *p* < 0.01 vs. control.
