# Supplementary figures and images for: Second generation androgen receptor antagonist, TQB3720 abrogates prostate cancer growth via AR/GPX4 axis activated ferroptosis
Source: Front Pharmacol. 2023 Jan 20;14:1110146. doi: 10.3389/fphar.2023.1110146 (PMC9895946; doi:10.3389/fphar.2023.1110146)

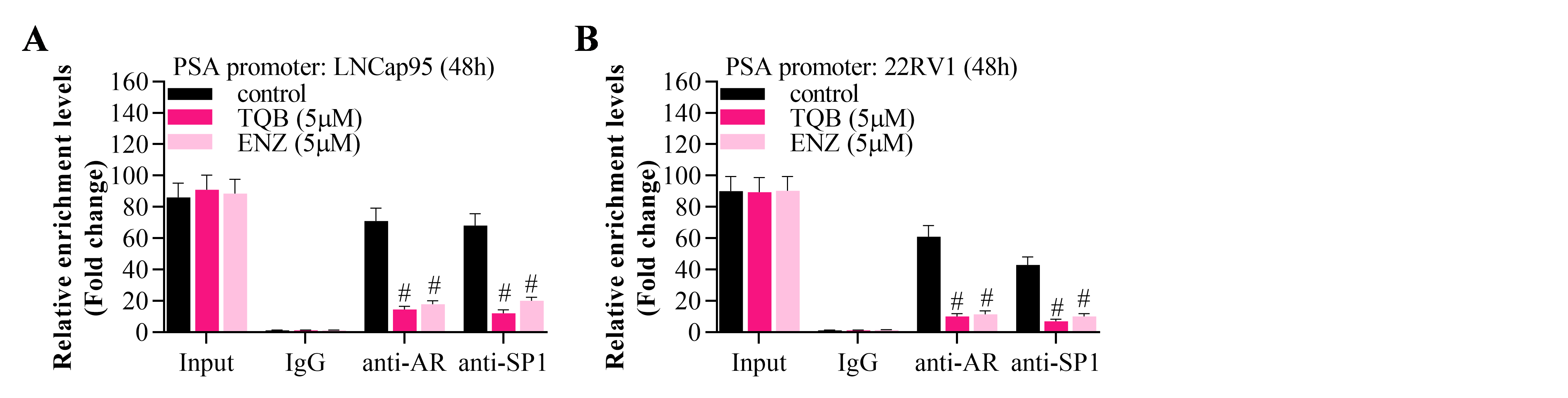

Supplement: Supplementary file 1 [file Image1.tif]
